# Optical Imaging for Biomedical Applications: From Rich Optical Contrast to Reliable Clinical Information

**DOI:** 10.3390/bioengineering13070837

**Published:** 2026-07-21

**Authors:** Johannes Dominikus Pallua

**Affiliations:** 1 Department of Orthopaedics and Traumatology, Medical University of Innsbruck, 6020 Innsbruck, Austria; johannes.pallua@i-med.ac.at; 2 Institute of Analytical Chemistry and Radiochemistry, University of Innsbruck, 6020 Innsbruck, Austria; 3 Institute of Pathology, Neuropathology and Molecular Pathology, Medical University of Innsbruck, Müllerstraße 44, 6020 Innsbruck, Austria; 4 Institute of Legal Medicine, Medical University of Innsbruck, Müllerstraße 44, 6020 Innsbruck, Austria

## 1. Introduction

Optical imaging has become an important enabling platform for biomedical research and clinical medicine. By exploiting the interactions of ultraviolet, visible, and infrared radiation with biological tissues, optical methods can provide structural, functional, molecular, and biochemical information without ionising radiation and, in many settings, with minimal disruption to the specimen or patient. The resulting technological toolbox spans conventional and computational microscopy, near-infrared spectroscopic imaging, hyperspectral imaging, Raman spectroscopy, multimodal image registration, and image-guided intervention.

This diversity represents a major strength, but it also exposes a central translational challenge: the information produced by an optical imaging system is only valuable when it can be acquired reproducibly, spatially aligned, interpreted robustly, and integrated into a clinically meaningful workflow.

The Special Issue “Optical Imaging for Biomedical Applications” was conceived to highlight advances across this translational pathway. Its six contributions encompass image-quality enhancement, physiological imaging, vibrational spectroscopy, surgical navigation, hyperspectral computer-aided diagnosis, and multimodal medical image registration [1,2,3,4,5,6]. Taken together, these studies demonstrate that progress in biomedical optical imaging increasingly depends not on a single instrument or algorithm, but on the coordinated development of acquisition hardware, calibration and registration procedures, computational analysis, validation strategies, and application-specific interpretation.

## 2. Improving Acquisition Quality and Extracting Physiological Information

A recurring limitation in optical imaging is that weak contrast, noise, and motion can obscure diagnostically relevant signals. Mahmoud et al. addressed this challenge in multispectral breast imaging by combining frame accumulation with a hybrid genetic algorithm–constriction factor particle swarm optimisation registration strategy [1]. Frame accumulation can improve the signal-to-noise ratio, but only when consecutive frames are sufficiently aligned. Camera jitter, respiratory motion, and patient movement may otherwise introduce image blur and reduce the benefits of averaging.

The proposed registration-before-accumulation framework therefore illustrates a principle with broad relevance: signal enhancement and motion correction should be treated as interdependent rather than separate processes. This is particularly important in dynamic, handheld, intraoperative, or bedside imaging, where acquisition conditions are less controlled than in laboratory-based experiments.

Leiva et al. focused on the physiological dimension of optical imaging by evaluating breath-holding as a practical stimulus for assessing peripheral oxygenation and flow using near-infrared spectroscopic imaging [2]. Their work demonstrates that a simple systemic manoeuvre can elicit measurable spatial and temporal changes in tissue oxygenation and perfusion-related parameters.

Such functional challenge tests are attractive because they move beyond static image interpretation and provide information on the dynamic response of biological tissues. However, clinical translation will require careful characterisation of interindividual variability, breathing protocols, tissue geometry, skin pigmentation, systemic haemodynamics, and instrument-specific sensitivity. Future studies should therefore establish standardised acquisition and reporting procedures and determine which derived parameters are sufficiently repeatable and robust for longitudinal monitoring or interventional applications.

## 3. Spectroscopy Across Scales: Chemical Specificity and Practical Deployment

Vibrational spectroscopy offers molecular and biochemical specificity that complements morphology- and perfusion-oriented optical imaging methods. Pallua et al. compared handheld and microscopic Raman spectroscopy for estimating the post-mortem interval of human bone [3]. This study illustrates the trade-off between analytical depth and practical deployability.

Raman microscopy enables spatially resolved measurements under controlled laboratory conditions, whereas handheld systems offer greater portability, faster acquisition, and the potential for use close to the site of examination. The comparison also emphasises that performance should be evaluated at the level relevant to the intended application. Strong separation in exploratory spectral models does not automatically guarantee robust classification of independent specimens. Differences in sampling area, fluorescence background, instrument response, acquisition conditions, and tissue heterogeneity may substantially influence analytical performance.

These considerations extend beyond forensic applications. Similar scale-dependent challenges are relevant to tumour-margin assessment, infection diagnostics, tissue-viability evaluation, and bedside monitoring. An important future priority is therefore the development of calibration-transfer and domain-adaptation strategies that enable predictive models to remain valid across instruments, operators, institutions, and acquisition geometries.

Reference materials, standardised preprocessing procedures, transparent reporting of spectral exclusion criteria, and validation at the patient, donor, or specimen level will be essential. Multimodal approaches may be particularly valuable: spatial optical imaging can identify regions of interest, while Raman or infrared spectroscopy can provide targeted biochemical confirmation.

## 4. Registration and Artificial Intelligence as Translational Infrastructure

The value of multimodal imaging depends critically on accurate spatial registration. Darzi and Bocklitz reviewed medical image registration across different modalities and summarised the persistent challenges associated with differences in spatial resolution, contrast mechanisms, geometric distortion, dimensionality, and tissue deformation [6].

These difficulties are amplified when optical data are combined with radiological images, histological sections, or intraoperative measurements. Registration errors may propagate into segmentation, quantitative biomarker extraction, treatment planning, and machine-learning labels. Registration accuracy should therefore be reported as a core performance variable rather than treated as an unreported preprocessing step.

Lee et al. demonstrated how deep learning can strengthen an established registration workflow for ear–nose–throat surgical navigation [4]. Their refinement step was incorporated between coarse conventional registration and iterative closest-point fine registration and resulted in improved registration accuracy compared with the conventional pipeline in phantom experiments.

Importantly, the proposed method was designed to improve rather than replace an established clinical workflow. This hybrid approach may facilitate clinical translation because data-driven models are introduced where they provide a measurable advantage, while deterministic and physically interpretable components preserve stability and compatibility with existing systems.

Nevertheless, this study also highlights several open questions that apply broadly to artificial intelligence in optical imaging. Models trained on simulated or single-centre datasets must be evaluated under realistic conditions that include physiological motion, tissue deformation, anatomical variability, uncommon pathologies, and device drift. Prospective clinical validation, uncertainty estimation, failure detection, and human-factor assessment are required before improved technical accuracy can be assumed to translate into improved patient outcomes.

Regulatory readiness will additionally require transparent preprocessing, traceable model development, version control, and a clearly defined intended clinical role for the algorithm.

## 5. Evidence Synthesis and the Need for Generalisable Diagnostic Studies

Leung et al. presented a systematic meta-analysis of computer-aided breast cancer detection using hyperspectral imaging [5]. The included studies collectively indicated promising diagnostic performance, particularly with respect to specificity and overall classification accuracy. However, the relatively small evidence base, heterogeneous wavelength ranges, varying analytical approaches, and limited geographical diversity constrain the generalisability of the available findings.

The analysis also illustrates why headline performance values must be interpreted within their methodological context. Different machine-learning approaches showed encouraging results, but no single algorithm consistently emerged as superior across all reported performance measures.

The next generation of diagnostic optical imaging studies should therefore prioritise multicentre, prospectively designed investigations over increasingly complex retrospective models trained on narrowly defined datasets. Data partitions must respect the patient, donor, specimen, and acquisition hierarchy to prevent information leakage.

External validation should include clinically representative disease prevalence, borderline cases, heterogeneous patient populations, and technically imperfect acquisitions. In addition to sensitivity, specificity, accuracy, and the area under the receiver operating characteristic curve, future studies should report calibration, uncertainty, subgroup performance, clinical utility, and the frequency and consequences of non-evaluable measurements.

By integrating methodological advances, application-oriented studies, and evidence synthesis, this Special Issue provides a cross-disciplinary perspective on the technical and translational requirements for reliable biomedical optical imaging.

## 6. Knowledge Gaps and Future Research Directions

Several common knowledge gaps emerge across the contributions to this Special Issue.

First, the field still lacks broadly accepted standards for optical image and spectral acquisition, preprocessing, quality control, and metadata reporting. Without such standards, differences between devices, centres, and laboratories may be difficult to distinguish from genuine biological variability.

Second, many optical biomarkers remain device- and protocol-dependent. Their physiological or pathological meaning should be established through mechanistic experiments and comparisons with validated reference methods, rather than solely through statistical associations.

Third, spatial and spectral heterogeneity must be addressed explicitly. Biological tissues consist of mixtures of cells, extracellular matrices, fluids, pigments, and pathological alterations across multiple spatial scales. Averaging may improve measurement stability but can remove diagnostically relevant focal information. Conversely, highly localised measurements may reveal subtle features but can be sensitive to sampling error. Adaptive acquisition strategies, spatially resolved uncertainty maps, and multiscale computational models may help reconcile these competing requirements.

Fourth, reproducibility and open science should become integral components of optical imaging system development. Shared benchmark datasets, raw-data availability where ethically and legally permissible, standardised phantoms, openly accessible analysis code, and detailed reporting of instrument settings would make comparisons between studies more meaningful. Privacy-preserving multicentre learning and federated analysis may enable broader validation while respecting restrictions on biomedical data sharing.

Finally, translation requires evaluation in the setting in which the technology is intended to be used. A technically successful laboratory prototype must also be sufficiently rapid, ergonomic, interpretable, maintainable, and economically justified. Future investigations should determine whether optical imaging information changes clinical decisions, improves patient outcomes, reduces invasive procedures, or increases workflow efficiency.

Co-design involving clinicians, pathologists, forensic experts, engineers, patients, industry partners, and regulatory specialists should begin early in technology development rather than after the completion of algorithmic or instrumental development.

## 7. Conclusions

The six papers collected in this Special Issue capture the breadth and convergence of contemporary biomedical optical imaging. They range from improving raw image quality and eliciting functional tissue responses to chemically specific spectroscopy, multimodal registration, surgical navigation, and evidence synthesis for computer-aided diagnosis.

Their common message is that optical contrast alone is insufficient. Meaningful biomedical impact emerges when acquisition, registration, computation, validation, and clinical interpretation are integrated into a coherent system.
